# The Leucine Catabolite and Dietary Supplement β-Hydroxy-β-Methyl Butyrate (HMB) as an Epigenetic Regulator in Muscle Progenitor Cells

**DOI:** 10.3390/metabo11080512

**Published:** 2021-08-04

**Authors:** Virve Cavallucci, Giovambattista Pani

**Affiliations:** 1 Fondazione Policlinico Universitario A. Gemelli IRCCS, 00168 Roma, Italy; 2 Institute of General Pathology, Università Cattolica del Sacro Cuore, 00168 Roma, Italy

**Keywords:** β-Hydroxy-β-Methyl Butyrate (HMB), dietary supplements, histone acetylation, HDACs, lysine β-hydroxybutyrylation (Kbhb), ketone bodies, butyrate, myoblasts, myotubes, sarcopenia

## Abstract

β-Hydroxy-β-Methyl Butyrate (HMB) is a natural catabolite of leucine deemed to play a role in amino acid signaling and the maintenance of lean muscle mass. Accordingly, HMB is used as a dietary supplement by sportsmen and has shown some clinical effectiveness in preventing muscle wasting in cancer and chronic lung disease, as well as in age-dependent sarcopenia. However, the molecular cascades underlying these beneficial effects are largely unknown. HMB bears a significant structural similarity with Butyrate and β-Hydroxybutyrate (βHB), two compounds recognized for important epigenetic and histone-marking activities in multiple cell types including muscle cells. We asked whether similar chromatin-modifying actions could be assigned to HMB as well. Exposure of murine C2C12 myoblasts to millimolar concentrations of HMB led to an increase in global histone acetylation, as monitored by anti-acetylated lysine immunoblotting, while preventing myotube differentiation. In these effects, HMB resembled, although with less potency, the histone deacetylase (HDAC) inhibitor Sodium Butyrate. However, initial studies did not confirm a direct inhibitory effect of HMB on HDACs in vitro. β-Hydroxybutyrate, a ketone body produced by the liver during starvation or intense exercise, has a modest effect on histone acetylation of C2C12 cells or in vitro HDAC inhibitor activities, and, unlike Butyrate and HMB, did not interfere with myotube formation in a myoblast differentiation assay. Instead, βHB dramatically increased lysine β-hydroxybutyrylation (Kbhb) of histone tails, an epigenetic mark associated with fasting responses and muscle catabolic states. However, when C2C12 cells were exposed to βHB in the presence of equimolar HMB this chromatin modification was drastically reduced, pointing to a role for HMB in attenuating ketosis-associated muscle wasting. In conclusion, while their mechanistic underpinnings remain to be clarified, these preliminary observations highlight novel and potentially important activities of HMB as an epigenetic regulator and βHB antagonist in muscle precursor cells, to be further explored in their biomedical implications.

## 1. Introduction

Muscle wasting occurs in several pathophysiological conditions from prolonged immobilization, aging and malnutrition to chronic inflammatory disorders and cancer, leading to the patient’s loss of independence and increased risk for falls, severe injury and death [1]. There is hardly any effective treatment, at the moment, for this debilitating condition, although several approaches based on nutritional supplementation [2], physical exercise [3], and hormonal therapy [4] have been proposed, with a varying degree of success [5].

Loss of muscle mass and strength (sarcopenia [6]) is the result of increased muscle protein catabolism, whose triggers and downstream signaling effectors have been in recent years, at least in part, elucidated. Upregulation of muscle-specific E3 ubiquitin ligases Atrogin-1 and Murf-1 is pivotal to enhanced proteasome-dependent proteolysis and atrophy of muscle cells [7]. Autophagy, another nutrient-regulated degradative process, is also involved in muscle mass maintenance although it would seem to play a double and opposite role according to age (myofiber atrophy in the young and maintenance of muscle mass during aging) [8]. Such “final common pathway” for muscle wasting is in turn governed by multiple metabolic (Insulin/IGF, mTOR/Akt) and pro-/anti-inflammatory (TNF alpha/IL-6, Myostatin) factors and signaling pathways, eventually converging on the transcriptional regulators FoxOs and NF-kB [9,10]. Thus, aberrant muscle degradation in sarcopenia appears to result from specific disease-triggered genetic programs [11], whose manipulation may hold the key for successful prevention and treatment of the disease.

β-Hydroxy-β-Methyl Butyrate (HMB) is a naturally occurring catabolite of the essential amino acid leucine widely used as a dietary supplement among athletes to improve muscle mass/strength and reduce muscle damage by resistance training [12]. Several controlled clinical studies have also shown a positive effect of HMB, especially in association with exercise, on lean body mass in age-related sarcopenia, raising interest towards the clinical potential and the mechanisms of action of this compound [13,14]. A number of reported biological effects of HMB, including (a) stimulation of muscle protein synthesis through the mTOR/S6 kinase and mTOR/4EBP1 anabolic cascades [15,16], (b) inhibition of proteasome-dependent proteolysis [17], (c) normalization of autophagy [18], and (d) sarcolemmal stabilization via the HMG-CoA reductase-dependent neo-synthesis of cholesterol, may all contribute to counteracting muscle wasting and promoting hypertrophic muscle growth [19,20]; however, a unitary mechanism of action underlying these seemingly diverse beneficial actions is still elusive.

An emerging trend in biomedical research has highlighted, in the last decade, the intricate connection between cell metabolism and epigenetics, whereby several metabolic intermediates act as regulators of different classes of chromatin-modifying enzymes, thus connecting deoxyribonucleic acid (DNA) accessibility and gene transcription with nutritional and environmental factors [21]. This applies, for instance, to the link between the Krebs cycle intermediates and DNA methylation/demethylation reactions [22], or the coupling of histone acetylation levels with cell energy status by the fluctuation of intracellular Acetyl-CoA and NAD+/NADH, acting as substrates/modulators of protein acetylases and deacetylases, respectively. Along similar lines, β-hydroxybutyrate (βHB), a ketone-body derived from fatty acid oxidation, acts both as a direct histone modifier (through lysine β-hydroxybutyrylation, Kbhb) [23,24] and a class I histone deacetylase (HDAC1 and 2) inhibitor [25], to orchestrate cellular gene reprogramming in response to fasting and oxidative stress.

Based on the structural similarity with βHB and the potent HDAC inhibitor (HDACi) Butyrate [26], we hypothesized that HMB might act as an epigenetic regulator of histone acetylation/acylation and gene transcription in muscle cells. This idea was further corroborated by the previous literature evidence pointing to a protective role of HDACi in muscle atrophy due to nutrient deprivation, denervation, and aging [27,28,29].

Thus, we set out to investigate the impact of HMB on histone acetylation and β-hydroxybutyrylation in muscle cells, choosing as a model a broadly used murine myoblast cell line (C2C12) amenable of terminal muscle differentiation in vitro.

## 2. Results

### 2.1. HMB Increases Global Histone Acetylation in C2C12 Murine Myoblasts

**Figure 1:**
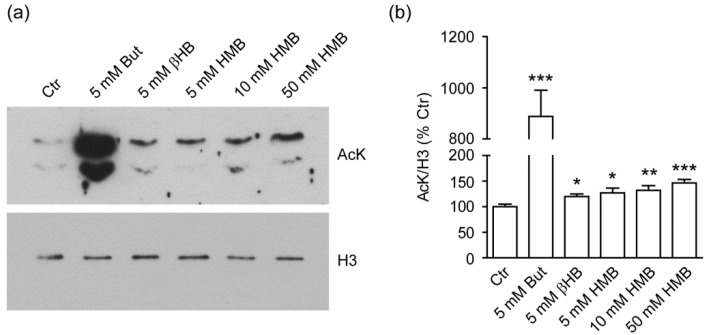
(**a**) Representative immunoblot analysis of acid-precipitated histones from undifferentiated C2C12 cells treated for 18 h with Butyrate (But), β-Hydroxybutyrate (βHB), or increasing concentrations of β-Hydroxy-β-Methyl Butyrate (HMB). An anti pan acetyl-lysine (AcK) antibody was used to assess global histone acetylation (upper panel); total histone H3 (lower panel) was used as loading control; (**b**) Densitometry of acetylated histone bands. Columns are mean ± SEM of OD values normalized for the untreated sample (% Ctr); *n* = 6 independent experiments. * *p* < 0.05; ** *p* < 0.01, *** *p* < 0.001 versus Ctr (untreated).

HMB is structurally related to both Butyrate, a product of bacterial fermentation, and the ketone body and endogenous lipid catabolite βHB (Supplementary Figure S1); these two short-chain fatty acids act as Class I HDACi and post-translational histone modifiers via lysine acetylation and β-hydroxybutyrylation [23,25], but a similar activity for HMB has not been reported far. To gain initial insight into the possible role of HMB as an HDACi, we exposed undifferentiated murine C2C12 myoblasts to increasing concentration (range 0–50 mM) of the compound for 18 h in standard growth medium (GM), and evaluated global lysine acetylation of acid-precipitated histones by anti acetylated-lysine (AcK) immunoblotting (Figure 1a). HMB increased histone acetylation over the baseline in a dose-dependent fashion, with a maximum at 50 mM (5 mM HMB *p* = 0.0230, 10 mM HMB *p* = 0.0068, 50 mM HMB *p* < 0.0001; Figure 1b). This effect resembled, although with much lower potency, that of Sodium Butyrate (*p* < 0.0001; Figure 1b). βHB impact on histone acetylation was also modest (*p* = 0.0220; Figure 1b) compared to Butyrate, at least at the single concentration tested, confirming that the reported effects of this compound as a strong HDACi may be cell- or context-dependent [30].

### 2.2. HMB and βHB Are Poor HDAC Inhibitors In Vitro

**Figure 2:**
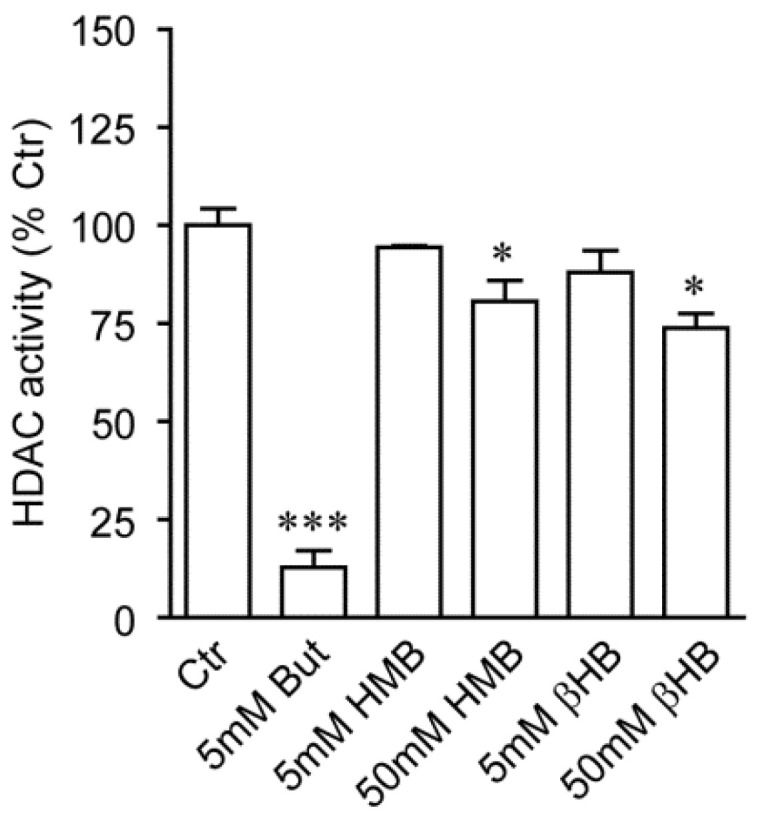
In vitro HDAC activity assay performed in the presence of the indicated concentrations of putative inhibitory compounds, or vehicle alone (Ctr). Deacetylation of the chromogenic substrate by HeLa cell crude nuclear extract was carried out for 20 min at RT and color development quantified by an ELISA microplate reader at 405 nm. Values are in absorbance units relative to the Ctr sample, set as 100% HDAC activity. Columns are mean ± SEM of three independent experiments. * *p* < 0.05; *** *p* < 0.001 versus Ctr. But: Butyrate; HMB: β-Hydroxy-β-Methyl Butyrate; βHB: β-Hydroxybutyrate.

Having observed an increase in global histone acetylation upon C2C12 cell treatment with HMB, we used a commercial HDAC inhibition assay kit to test whether this compound inhibits HDACs in a cell-free reaction (Figure 2). Sodium Butyrate and βHB were also assessed, alongside HMB. As revealed by spectrophotometric quantitation, deacetylation of the chromogenic substrate (COLOR DE LYS^®^, Enzo Life Sciences) by HDACs (prevalently HDAC 1 and 2) enzymes present in crude HeLa cell nuclear extracts was drastically inhibited by 5 mM Butyrate (*p* = 0.0001; Figure 2), to an extent comparable to the manufacturer-provided control inhibitor (Trichostatin). Conversely, HMB and βHB were largely ineffective at 5 mM (and 10 mM, data not shown), with a slight decrease in substrate deacetylation appearing for both compounds only at a tenfold higher concentration (50 mM HMB *p* = 0.0454, 50 mM βHB *p* = 0.0217; Figure 2). Of note, evidence against βHB acting as an HDACi has been recently reported [30].

These results do not support the idea that altered histone acetylation levels observed in response to HMB are due to a direct inhibitory role of HMB on HDACs.

### 2.3. Stage-Specific Effect of HMB on Myogenic Differentiation of C2C12 Cells

**Figure 3:**
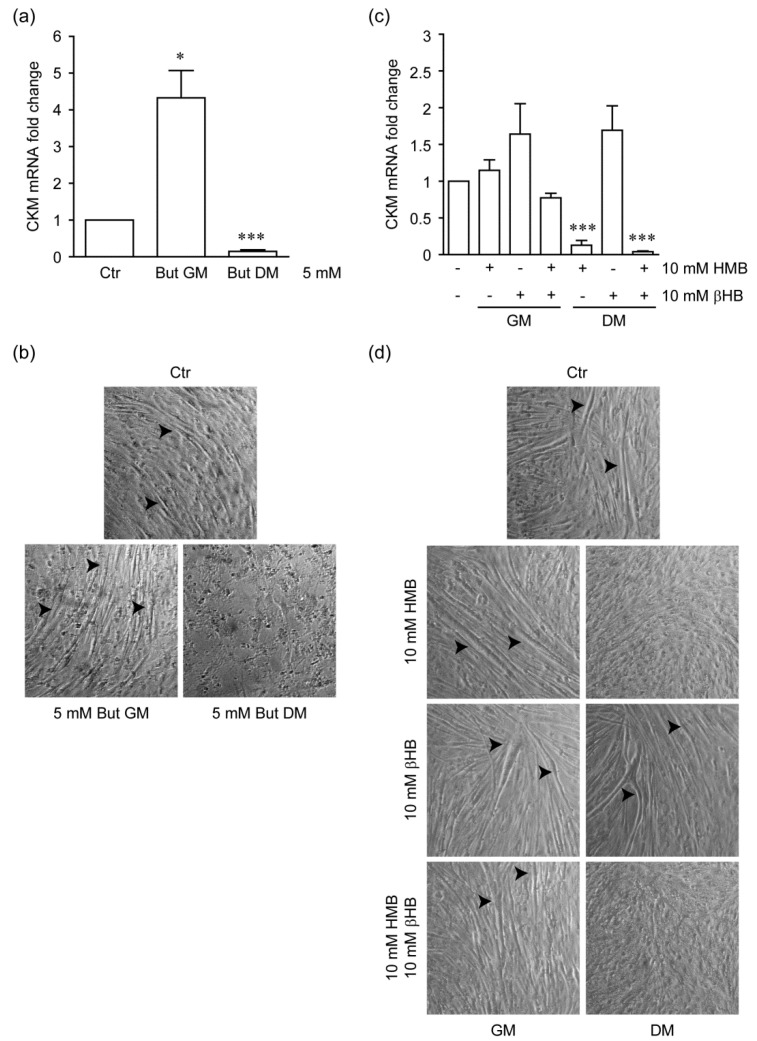
(**a**) Real-Time qPCR analysis of Muscle Creatine Kinase (CKM) mRNA expression in C2C12 cells exposed to Butyrate (But) at different stages of differentiation. GM: growth medium; DM: differentiation medium. Values are relative to cells undergoing the standard differentiation protocol without Butyrate (Ctr). Columns indicate mean ± SEM. * *p* < 0.05; *** *p* < 0.001 versus Ctr (*n* = 3 independent experiments, paired *t*-test). (**b**) Representative microphotographs of cultures differentiated under the conditions as in panel a. Typical elongated syncytia (myotubes) are indicated by arrowheads. (**c**) Effect of β-Hydroxy-β-Methyl Butyrate (HMB) and β-Hydroxybutyrate (βHB) on CKM mRNA expression in differentiating C2C12 cultures. Compounds were given separately or in combination, in GM or in DM as indicated. Values are relative to untreated Ctr. *** *p* < 0.001 versus Ctr (*n* = 3 independent experiments, paired *t*-test). (**d**) Representative microphotographs of end-differentiated cultures from panel c (DM, day 7). Note the lack of myotubes (arrowheads) in samples exposed to HMB alone or in combination with βHB.

We used a stage-specific experimental paradigm [31] to evaluate the functional relevance of HMB-induced histone acetylation compared to Butyrate and βHB. Cells were given HMB, Butyrate or βHB either in growth medium (GM) for 48 h before the change to differentiation medium (DM), or directly in DM for 7 days. As expected [31], early cell exposure to Butyrate enhanced myogenesis, assessed by the expression of Muscle Creatine Kinase (*Ckm*), a gene upregulated during the late stages of muscle differentiation [31] (Supplementary Figure S2 and Figure 3a, *p* = 0.0111) and the number/size of multinucleated myotubes (Supplementary Figure S2 and Figure 3b); instead, treatment of C2C12 cells with Butyrate in DM medium profoundly impaired differentiation and induced cell death (Figure 3a,b; *p* < 0.0001). Commensurate with its limited potency in inducing global histone acetylation, HMB had a modest, if any, effect in priming proliferating cells to myogenic differentiation. However, similar to Butyrate, HMB almost completely prevented terminal differentiation (as monitored by *Ckm* expression levels) and myotube formation in DM (Figure 3c,d; *p* < 0.0001). Interestingly, such an inhibitory effect was not observed in cultures differentiated in the presence of βHB; instead, a slight enhancement of muscle gene expression was inconsistently noted in cells treated with the ketone body before or after the switch to differentiative conditions (Figure 3c,d). Of note, βHB did not revert the inhibitory effect of HMB when the two compounds were applied together in DM (Figure 3c,d; *p* = 0.0001).

These findings highlight significant differences in the C2C12 myogenic differentiation paradigm among the tested compounds, pointing to overlapping but distinct biochemical actions.

### 2.4. HMB Inhibits Histone β-Hydroxybutyrylation by βHB

**Figure 4:**
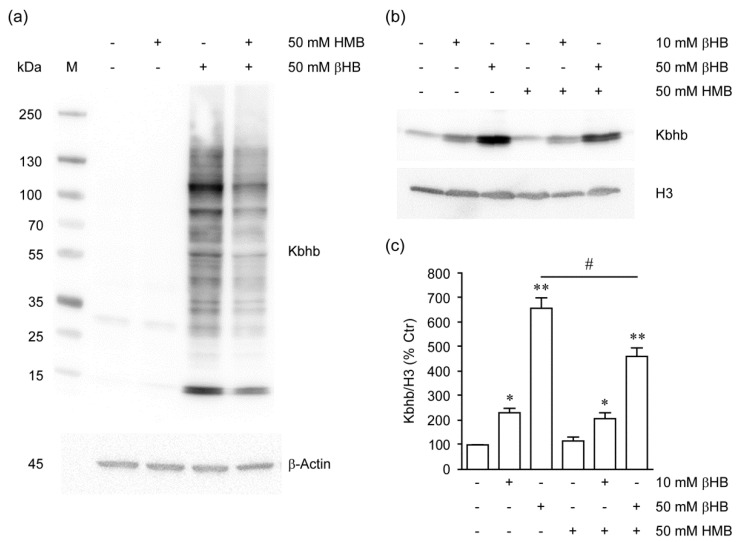
(**a**) Immunoblot analysis of total protein lysates from undifferentiated C2C12 cells exposed for 18 h to β-Hydroxybutyrate (βHB), β-Hydroxy-β-Methyl Butyrate (HMB) or the combination thereof. Protein hydroxybutyrylation on lysine residues (Kbhb) was detected with a pan anti Kbhb antiserum; β-Actin was used as loading control. (**b**) Representative immunoblot of acid-precipitated histones from C2C12 cells treated as indicated and probed with an anti-Kbhb antibody (upper panel); the amount of total H3 is shown as protein loading control (lower panel); (**c**) Densitometry of Kbhb histone bands from multiple (*n* = 3) experiments under the indicated treatments. Columns are mean ± SEM of OD values normalized for the untreated sample (% Ctr). * *p* < 0.05; ** *p* < 0.01 versus Ctr; # *p* < 0.05 between the two treatments.

To further pursue the hypothesis of HMB acting as an epigenetic regulator on muscle cells, we asked whether HMB may interfere with lysine β-hydroxybutyrylation (Kbhb), a specific histone post-translational modification (PTM) promoting gene expression in response to starvation-responsive metabolic pathways [23,24,30]. Expectedly, exposure of undifferentiated C2C12 cells to 5–50 mM βHB elicited a marked accumulation of hydroxybutyrylated proteins over the entire spectrum of the molecular weight range, as revealed by immunoblotting of cell homogenates with a polyclonal anti pan Kbhb antiserum (Supplementary Figure S3 and Figure 4a). Additionally, βHB dose-dependently increased Kbhb reactivity of acid-precipitated histones (Figure 4b,c; 10 mM βHB *p* = 0.0184, 50 mM βHB *p* = 0.0058); Butyrate, even at the highest concentration used (50 mM), did not elicit this PTM in total cell lysates (Supplementary Figure S4). HMB was equally ineffective in promoting Kbhb per se, but strongly reduced the effect of βHB on total protein lysates (Figure 4a and Supplementary Figure S3) and isolated histones (Figure 4b,c; *p* = 0.0168). The same effect on Kbhb was also observed in whole homogenates from HEK 293T cells exposed to βHB and/or HMB (Supplementary Figure S5).

Although the level at which such inhibitory effect occurs cannot be determined in the above experimental settings, these findings clearly show altered protein and histone Kbhb levels in muscle cells exposed to pharmacologically relevant concentrations of HMB.

## 3. Discussion

The mechanisms underlying the beneficial effects of HMB on muscle mass and strength in sports training and pathologic states remain poorly understood. Elucidating the cellular and molecular interactions whereby this endogenous metabolite and dietary supplement impact muscle health may pave the way to novel pharmacological strategies against sarcopenia and frailty.

The present work addresses the novel hypothesis that HMB shares functional properties with the chemically related molecules Butyrate and βHB, two compounds known to act as epigenetic drugs and regulators of gene expression via chromatin acetylation/acylation of histone tails. This idea appeared particularly attractive in view of the emerging role of histone deacetylases in muscle atrophy following denervation and aging, and the potential of small-molecule HDAC inhibitors as muscle-enhancing drugs [28,29].

Our initial analysis confirmed that HMB, in the millimolar range (5–50 mM), increases global histone acetylation of C2C12 cells, although with much lower potency compared to Butyrate. Importantly, however, we failed to document HDAC inhibitory activity of HMB in an in vitro enzyme assay, which raises doubts on this compound acting, unlike Butyrate, directly on histone deacetylases. It is still possible that, in intact cells, HMB reduces HDAC activity indirectly, by interacting with a co-factor missing in a cell-free assay, or by lowering HDACs expression and hence catalytic action. Alternatively, HMB may favor histone acetylation by targeting histone acetyl-transferase (HAT) activities; in particular, we suggest that a rise in the intranuclear levels of the HAT co-substrate Acetyl-CoA, to which (together with acetoacetate) HMB is ultimately catabolized in the cell cytosol [32], may contribute to effects displayed in Figure 1a,b.

Irrespective of the mechanisms, the question arises as to whether and to which extent the modest increase in global histone acetylation detected in HMB-treated C2C12 cells is biologically relevant to the compounds’ beneficial effects on muscle health. Myoblast differentiation studies displayed in Figure 3c,d clearly show that HMB in part recapitulates the stage-specific effects of Butyrate (Figure 3a,b), i.e., promotion of muscle-specific gene expression when given before the differentiation switch, and a substantial inhibitory effect on myotube formation and muscle-specific gene expression if continuously present in the differentiation medium [31]. Since these complex actions have been convincingly correlated with Butyrate-induced hyperacetylation of histones surrounding the binding sites of MyoD, a major muscle-regulatory factor, it is conceivable that increased histone acetylation by HMB contributes to its effects on myoblast differentiation in vitro, and by extension to HMB properties in vivo. More detailed studies employing chromatin immunoprecipitation to address histone acetylation at specific genomic regions will hopefully further corroborate this view (see below).

**Figure 5:**
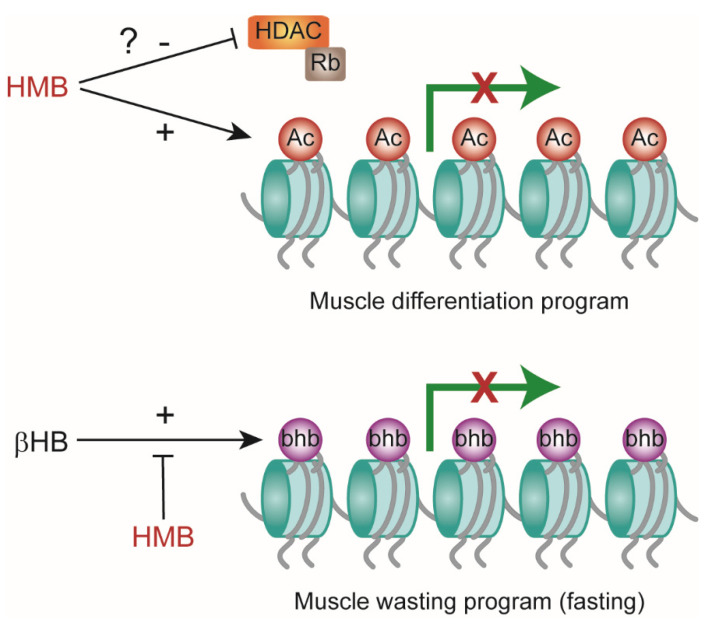
Schematic representation of β-Hydroxy-β-Methyl Butyrate (HMB) and β-Hydroxybutyrate (βHB) effects on histones modification and gene expression. Ac: acetylation; bhb: β-hydroxybutyrylation. + (plus) denotes activation/induction; − (minus) denotes inhibition/blockade.

Our data also suggest that HMB effects on muscle cell differentiation may involve additional histone PTMs; in fact, we have shown that HMB interferes with total protein and histone hydroxybutyrylation by βHB. This newly described protein lysine modification and histone mark mediates regulation of gene expression in cultured cells exposed to βHB and, most importantly, metabolic genetic reprogramming in response to fasting or diabetes-associated ketogenesis [23]. In our setting, global histone hydroxybutyrylation was markedly and dose-dependently increased by βHB, while the effects of this compound on histone acetylation was subtle and, unlike previously reported [25], marginal HDAC inhibitory activity could be detected in a cell-free enzyme assay (Figure 2). Moreover, βHB did not inhibit myotube formation (Figure 3c,d), as it would be expected from an HDACi [31], in the C2C12 differentiation model (Figure 3a,b). HMB, even at the highest concentration tested (50 mM), was unable to induce protein or histone Kbhb; however, HMB reduced Kbhb accumulation elicited by hydroxybutyrate. Mechanistically, this novel observation may have several explanations. HMB may compete with βHB at the level of the substrate, by targeting the same lysine residues with a different acyl group (hydroxymethylglutarylation representing a possible candidate [33]) that is not recognized by the anti Kbhb antiserum. In alternative, HMB may inhibit the (yet to be characterized) βOH butyryl-CoA transferase so as to reduce the levels of Kbhb, without an alternative PTM being simultaneously created. Finally, the possibility of competition for transmembrane transport between these two structurally similar compounds should not be overlooked. The HMB-induced PTMs are summarized in Figure 5.

While a mechanism is still elusive, the observation that HMB antagonizes βHΒ-dependent chromatin marking and hence metabolic gene regulation, if confirmed in more physiological settings, may entail far-reaching implications for muscle biology. Since ketone bodies participate in body metabolic rewiring in fasting and diabetes, two conditions associated with muscle wasting, HMB, by attenuating histone Kbhb and the associated catabolic genetic response, may help to maintain muscle mass in conditions of low insulin signaling. Of note, similar to diabetes, age-dependent sarcopenia may as well be viewed as a form of muscle-selective resistance to insulin [34].

This idea is especially attractive when considering that HMB is endogenously generated during leucine catabolism in muscle cells, and may physiologically operate in mitigating protein degradation in muscle degradative states. In keeping with this view, conversion of leucine to HMB is required for stimulation of protein synthesis in rat myotubes [35]. Thus, in a broader perspective, our findings may add HMB to the list of endogenous intermediates linking metabolic and epigenetic states in muscle cells.

The main limitation of the present study resides in its preliminary nature. While the reported observations of increased histone acetylation and reduced hydroxybutyrylation by HMB are novel and potentially significant, the mechanistic underpinnings still need to be elucidated. In particular, more sophisticated proteomic approaches are warranted to comprehensively profile HMB-related histone PTMs, clarifying whether the observed changes in AcK and Kbhb involve overlapping or distinct lysine residues and are causally linked to each other. Moreover, chromatin immunoprecipitation assays are critically required to investigate HMB’s epigenetic effects either at specific genomic sites relevant to muscle development and metabolism, or possibly genomewide (Chip-Seq).

Another concern deals with the elevated concentrations of metabolites (millimolar range) used in the study. However, although HMB plasma levels have been reported in the micromolar range even after supplementation [32,36], the intracellular or even subcellular (nuclear?) concentration of the endogenous metabolite is hard to predict and may vary according to muscular functional states. Future in vivo studies on animal muscle tissue could help to clarify this aspect and contribute to understanding the mechanisms of HMB action.

Notwithstanding the above limitations, the presented data offer novel insights into the mechanism whereby HMB exerts its beneficial effects on muscle mass and strength, and may guide the selection or development of more effective pharmacological tools against frailty and sarcopenia.

## 4. Materials and Methods

### 4.1. Cell Culture

The C2C12 mouse myoblast cell line (obtained from American Type Culture Collection, ATCC, Manassas, VA, USA, catalog code CRL-1772™) was maintained in growth medium (GM) containing Dulbecco’s Modified Eagle’s Medium (DMEM) high glucose with Sodium Pyruvate with l-Glutamine, MEM non Essential Amino Acids, 100 U/mL penicillin/streptomycin, and 20% fetal bovine serum (FBS) in a humidified incubator at 37 °C and 5% CO_2_. For myogenic differentiation cells were plated on gelatin-coated plates (0.2% gelatin from bovine skin) at 5 × 10^3^ cells/cm^2^. The GM was replaced after 48 h with differentiation medium (DM) consisting of DMEM high glucose (with Sodium Pyruvate with l-Glutamine), MEM non Essential Amino Acids, 100 U/mL penicillin/streptomycin, and 5% horse serum (HS). Cells were cultured for 7 days and cell morphology monitored by light microscopy.

HEK 293T cells (obtained from American Type Culture Collection, ATCC, catalog code CRL-3216), a highly transfectable derivative of human embryonic kidney 293 cells expressing a temperature-sensitive SV40 T-antigen, were routinely propagated in growth medium containing DMEM high glucose, 2 mM Glutamine, 1 mM Sodium Pyruvate, MEM non Essential Amino Acids, 100 U/mL penicillin/streptomycin, and 10% FBS) in a humidified incubator at 37 °C and 5% CO_2_. Cells were seeded at 10^5^/cm^2^ in 12 multiwell plates, 16–24 h before stimulation in regular growth medium.

Cells were treated for 18 h with sodium butyrate (Sigma-Aldrich, St. Louis, MO, USA), calcium β-hydroxy-β-methylbutyrate (Galeno Srl, Comeana, Italy), and dl-β-hydroxybutyric acid (Sigma-Aldrich), each diluted from a 500 mM sterile stock solution in PBS.

### 4.2. Histone Extraction

Histone extraction was performed as previously described [25], with minor modifications due to the biological starting material (mouse tissue vs. cultured cells). Briefly, at the end of treatment cells were harvested and washed with ice-cold PBS supplemented with 5 mM sodium butyrate. The cell pellet was resuspended in Triton Extraction Buffer (TEB: PBS containing 0.5% Triton X-100, 2 mM phenylmethylsulfonyl fluoride, 0.02% sodium azide), incubated on ice for 10 min with gentle stirring and centrifuged at 6500× *g* for 10 min at 4 °C. Pellet was washed with TEB and resuspended in 0.2 N HCl. Following overnight acid extraction at 4 °C samples were centrifuged at 6500× *g* for 10 min at 4 °C and supernatant collected for Immunoblotting analysis.

### 4.3. Total Lysates and Immunoblotting

After treatment, adherent cells were washed once with cold PBS to remove serum proteins and lysed on ice with 1× SDS Lemmli sample buffer (50 mM Tris-HCl pH 6.8, 5% β-mercaptoethanol, 10% glycerol, 1% sodium dodecyl sulfate (SDS), 1.5 mM bromophenol blue). Samples were sonicated for 10 sec and then heated to 95 °C for 10 min before loading.

Proteins were applied to SDS-PAGE and electroblotted onto a nitrocellulose membrane. Immunoblotting analysis was performed using a chemiluminescence detection kit. The relative levels of immunoreactivity were determined by using the NineAlliance™ software (Uvitec, Cambridge, UK).

Primary antibodies were the following: Acetylated-Lysine (Cell Signaling Technology, Danvers, MA, USA; #9441), Histone H3 (Cell Signaling Technology, Danvers, MA, USA; #3638), β-Actin (Cell Signaling Technology, Danvers, MA, USA; #3700), β-Hydroxybutyryllysine (PTM Biolabs, Chicago, IL, USA; #PTM-1201).

### 4.4. Colorimetric Assay of Histone Deacetylase Activity

Histone deacetylase (HDAC) activity was measured by the COLOR DE LYS^®^ HDAC colorimetric activity assay kit (Enzo Life Sciences, Farmingdale, NY, USA; #BML-AK501-0001) according to the manufacturer’s instructions.

### 4.5. Quantitative Real-Time PCR

RNA was extracted from cell cultures by the Direct-zol RNA Miniprep kit (Zymo Research, Irvine, CA, USA; #R2052), according to the manufacturer’s instructions. The amount and purity of RNA were determined by NanoDrop™ (Thermo Fisher Scientific, Whaltam, MA, USA). cDNA was obtained by RNA retro-transcription using the SensiFAST™ cDNA Synthesis Kit (Meridian Bioscience, Cincinnati, OH, USA). Real-time PCR was performed with SensiFAST™ SYBR^®^ No-ROX Kit (Meridian Bioscience, Cincinnati, OH, USA) in a CFX96 qPCR Instrument (Bio-Rad, Hercules, CA, USA).

The following oligonucleotide primers were used:

Tata binding protein (TBP) forward CTACCGTGAATCTTGGCTGTAAAC and reverse: AATCAACGCAGTTGTCCGTGGC;

Creatin kinase, muscle (CKM) forward: GGCTTCACTCTGGACGATGTCA and reverse: CCTTGAAGACCGTGTAGGACTC.

### 4.6. Data Analysis and Statistics

The values are shown as % of the untreated control (mean ± SEM). Data were collected from at least three independent experiments, and means were compared by two-tailed Student’s *t*-test for unpaired data (GraphPad Prism software, version 5.01), unless otherwise indicated. Differences were considered significant at *p* < 0.05.

## 5. Conclusions

Taken together our results highlight novel epigenetic mechanisms of action of HMB as an anti-sarcopenic agent; additionally, they provide a rationale for chemical modifications aimed at improving the biological activity of this compound and encourage further research on epigenetic drugs for the prevention and treatment of muscle wasting.

## Data Availability

The data presented in this study are available in article.

## Supplementary Materials

The following are available online at https://www.mdpi.com/article/10.3390/metabo11080512/s1, Figure S1: Structure of Butyric acid, β-Hydroxybutyric acid, and β-Hydroxy-β-Methylbutyric acid, Figure S2: Muscle Creatine Kinase (CKM) mRNA is upregulated in C2C12 differentiated cells, Figure S3: C2C12 myoblast exposure to β-Hydroxybutyrate (βHB) leads to lysine-hydroxybutyrylation in total cell lysates, Figure S4: Butyrate has no effect on lysine-hydroxybutyrylation in total C2C12 cell lysates, Figure S5: Inhibitory effect of β-Hydroxy-β-Methyl Butyrate (HMB) on total lysine-hydroxybutyrylation in HEK 293T cells.
