# Supplementary material for: The Leucine Catabolite and Dietary Supplement β-Hydroxy-β-Methyl Butyrate (HMB) as an Epigenetic Regulator in Muscle Progenitor Cells
Source: Metabolites. 2021 Aug 4;11(8):512. doi: 10.3390/metabo11080512 (PMC8399199; doi:10.3390/metabo11080512)
Supplement: Supplementary file 1 [file metabolites-11-00512-s001.zip › metabolites-1321833-supplementary.pdf]

metabolites

MDPI

*Communication*

**The leucine catabolite and dietary supplement Hydroxy-Methyl Butyrate (HMB) as an epigenetic regulator in muscle progenitor cells**

**Virve Cavallucci and Giovambattista Pani**

# Supplementary Figures

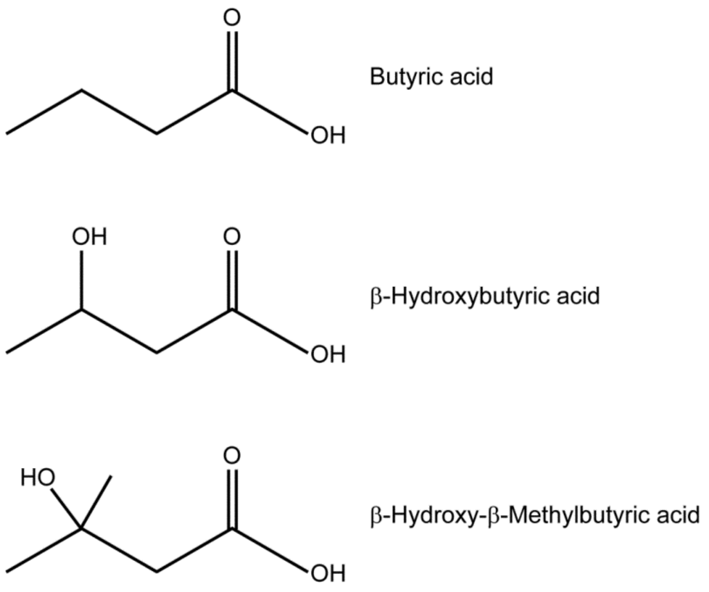


**Figure S1.** Structure of Butyric acid, β-Hydroxybutyric acid, and β-Hydroxy-β-Methylbutyric acid.

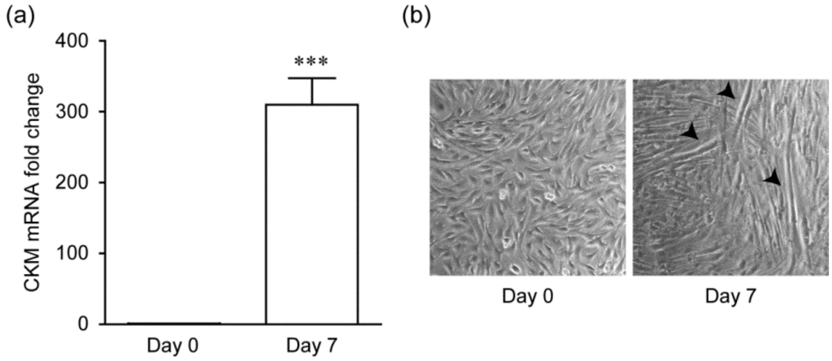


**Figure S2.** Muscle Creatine Kinase (CKM) mRNA is upregulated in C2C12 differentiated cells. (**a**) Real-Time qPCR analysis of mRNA expression in C2C12 myoblasts before differentiation induction (day 0) and at the end of a standard differentiation protocol (day 7). Values are relative to undifferentiated cells (day 0). Columns indicate mean ± SEM. ***$p<0.001$ versus Ctr (n=6 independent experiments, paired t test). (**b**) Representative microphotographs of cultures before (day 0) and after (day 7) differentiation. Typical elongated syncytia (myotubes) are indicated by arrowheads.

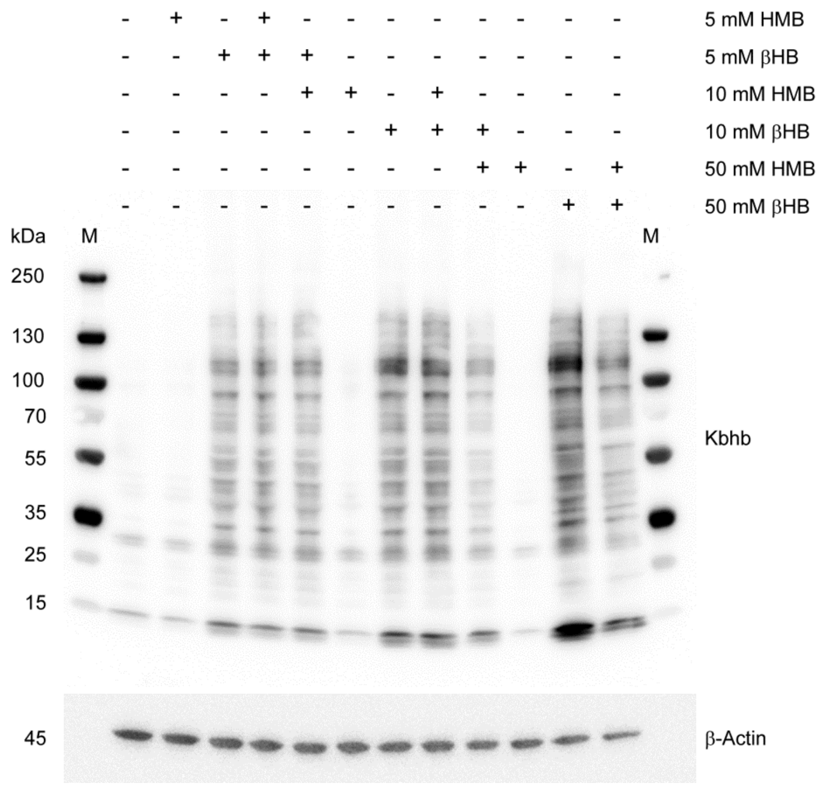


**Figure S3.** C2C12 myoblast exposure to β-Hydroxybutyrate (βHB) leads to lysine-hydroxybutyrylation in total cell lysates. Representative immunoblot analysis of total protein lysates from undifferentiated C2C12 cells exposed for 18 hours to different concentration of βHB, β-Hydroxy-β-Methyl Butyrate (HMB) or the combination thereof as indicated. Protein hydroxybutyrylation on lysine residues (Kbhb) was detected with an anti-Kbhb antibody; β-Actin is shown as loading control.

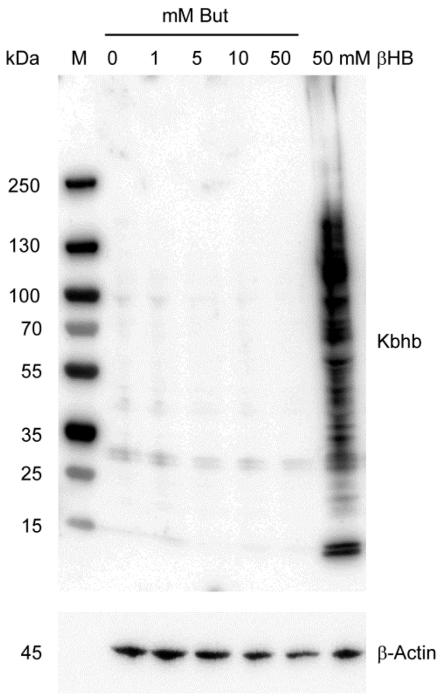


**Figure S4.** Butyrate has no effect on lysine-hydroxybutyrylation in total C2C12 cell lysates. Representative immunoblot analysis of total homogenates from undifferentiated C2C12 cells exposed for 18 hours to increasing concentration of Butyrate (But). Protein hydroxybutyrylation on lysine residues (Kbhb) was detected with an anti-Kbhb antibody; β-Actin is shown as loading control. M: molecular weight marker.

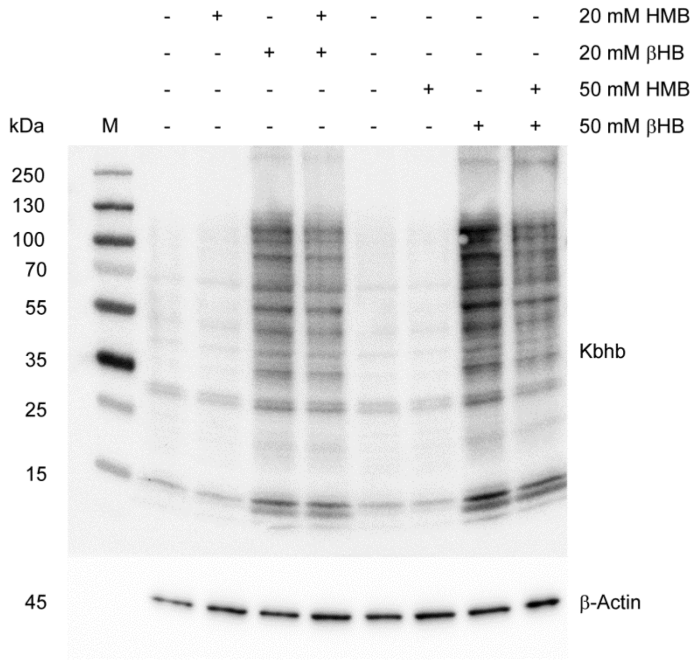


**Figure S5.** Inhibitory effect of β-Hydroxy-β-Methyl Butyrate (HMB) on total lysine-hydroxybutyrylation in HEK 293T cells. Representative immunoblot analysis of whole protein lysates from HEK 293T cells exposed for 18 hours to different concentration of β-Hydroxybutyrate (βHB), HMB or the combination thereof as indicated. Protein hydroxybutyrylation on lysine residues (Kbhb) was detected with an anti-Kbhb antibody; β-Actin is displayed as loading control. M: molecular weight marker.
